# Extracellular MicroRNAs: A Stable and Diverse Source of Transcriptional Control

**DOI:** 10.3390/biom16060787

**Published:** 2026-05-27

**Authors:** Megan I. Mitchell, Olivier Loudig

**Affiliations:** 1 Center for Discovery and Innovation, Hackensack Meridian Health (HMH), Nutley, NJ 07110, USA; megan.mitchell@hmh-cdi.org; 2 Oncology Department, Georgetown University, Washington, DC 20007, USA

**Keywords:** microRNA, intracellular, extracellular, isomiRs, Dicer, Drosha, seed sequence, mRNA, deregulation, cancer, extracellular vesicles

## Abstract

MicroRNAs (miRNAs) are a highly conserved class of small (19–25 nucleotides) non-coding RNAs that play critical roles in post-translational gene regulation. Dysregulation of miRNA expression has been widely implicated in the development and progression of numerous diseases, particularly cancer, positioning them as promising candidates for diagnostic and prognostic applications. In parallel, miRNAs are frequently detected in extracellular vesicles (EVs), where they contribute to intercellular communication and have emerged as attractive non-invasive biomarkers. Importantly, EV-associated miRNA profiles do not always directly mirror intracellular miRNA abundance. While altered cellular expression can influence EV-miRNA content, selective and regulated sorting mechanisms also actively shape EV cargo composition. These include sequence- and motif-based recognition elements (such as EXOmotifs), RNA-binding proteins (including hnRNPA2B1, YBX1, and SYNCRIP), and lipid-associated pathways such as ceramide-dependent mechanisms. Together, these processes enable the preferential packaging of specific miRNAs into EVs, independent of their relative cellular expression levels. This review therefore integrates both perspectives: it summarizes current evidence supporting dysregulated miRNAs detected in EVs as disease-associated biomarkers and critically examines the molecular mechanisms governing miRNA sorting into EVs. By clarifying the interplay between cellular miRNA dysregulation and active EV loading processes, we highlight the complexity underlying EV-miRNA signatures and underscore the need for standardized mechanistic frameworks to improve their translational utility in cancer diagnostics and beyond.

## 1. Introduction

MicroRNAs (miRNAs) are a highly conserved class of small (19–25 nucleotides), non-coding RNA molecules that serve as critical post-transcriptional regulators of gene expression [1,2,3]. Approximately 2000 unique miRNA sequences have been identified in humans, with expression tightly regulated across all cell types and their developmental stages [4]. Functioning as important regulators, miRNAs primarily exert their influence by binding to complementary sequences within the 3′ untranslated regions (3′UTRs) of target messenger RNAs (mRNAs). This results in translational repression or mRNA degradation and thus negative regulation of gene expression [5]. The precise impact of a given miRNA depends on the degree of sequence complementarity with its mRNA target but also on the collective action of multiple miRNAs acting in concert [6]. A single miRNA can target numerous mRNAs, and conversely, individual mRNAs can be regulated by multiple distinct miRNAs [7]. This redundancy and crosstalk give rise to intricate gene regulatory networks, allowing for precise control over cellular pathways [8,9]. Dysregulation in miRNA expression has been strongly linked to the development and progression of numerous diseases, most notably cancer [10]. Aberrant miRNA profiles are now recognized as hallmarks of cancer [11], with well-documented roles in tumor initiation, progression, metastasis, and resistance to therapy [12,13,14]. Depending on their targets and the cellular context, miRNAs can act either as oncogenes (commonly referred to as “oncomiRs”) or as tumor suppressors [15,16]. By modulating expression programs involved in angiogenesis, epithelial–mesenchymal transition (EMT), and cancer stem cell maintenance, miRNAs play a central role in orchestrating the tumor microenvironment and disease trajectory [17,18]. Importantly, the identification of disease-specific miRNA expression patterns has highlighted their promise as biomarkers for disease diagnosis and prognosis and for the prediction of therapeutic response [19]. However, miRNAs do not solely exert their regulatory effects intracellularly. Over the past decade, research has shown that they can also be selectively packaged into extracellular vesicles (EVs), allowing for their stable export and transport through the local microenvironment and bloodstream. In this way, miRNAs can regulate gene expression at a distance by targeting mRNAs in recipient cells [20]. Circulating EV-bound miRNAs can be detected in various bodily fluids, including blood, saliva, urine, tears, sweat, bronchoalveolar lavage fluid (BALF), exhaled breath condensates (EBCs), and many other biofluids [21,22,23]. The robust phospholipid bi-layer of EVs preserves packaged miRNAs’ integrity during transport, which enables post-transcriptional control/communication and positions EV-associated miRNAs as important mediators of cell-to-cell signaling. While miRNAs are among the most extensively studied regulatory molecules, a critical gap remains in integrating intracellular miRNA biology with EV-mediated transport mechanisms. Current studies frequently decouple miRNA biogenesis, isoform diversity, and target regulation from selective EV packaging and intercellular communication, limiting the biological interpretation of circulating miRNA profiles. Compounding this issue, a growing reproducibility crisis, driven by inconsistent EV isolation methods and the lack of standardized normalization strategies, has hindered the validation and clinical translation of EV-associated miRNA biomarkers. Therefore, this review aims to bridge these gaps by providing an integrated framework that connects intracellular miRNA regulation with EV-mediated transport, while critically evaluating the technical and analytical barriers limiting reproducibility and clinical application.

## 2. MiRNA Nomenclature, Structure, and Mode of Action

**Figure 1:**
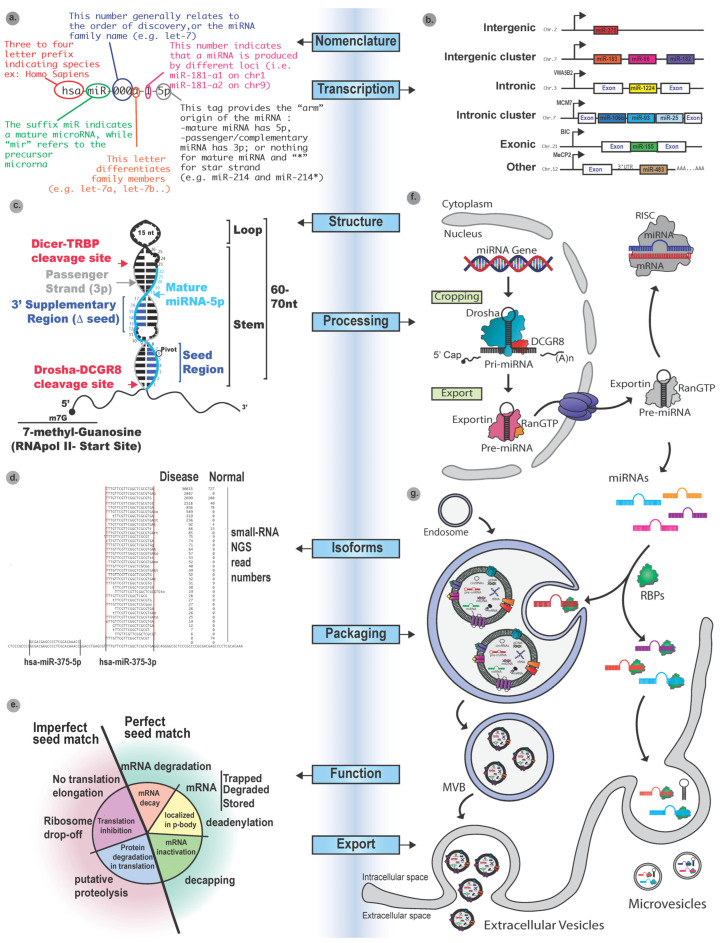
Comprehensive overview of microRNA biogenesis, structure, function and their packaging and export within extracellular vesicles. (**a**) Standardized miRBase nomenclature of human miRNAs (e.g., hsa-miR-000a-1-5p) includes a species prefix (e.g., hsa for Homo sapiens), numerical identifiers based on discovery order or miRNA families, alphabetical characters to denote closely related family members, locus-specific suffixes (e.g., −1, −2), and strand designations (5p for the guide strand, 3p for the passenger strand). (**b**) Genomic origin of miRNA genes, which are located in intergenic regions as single genes or clusters, within introns or exons of protein-coding genes as single genes or clusters, or within single genes or polycistronic clusters. Shown are examples such as miR-375 (intergenic on chromosome 2), the miR-183/96 cluster (intergenic cluster on chromosome 7), miR-1224 (intronic within the VWA5B2 gene on chromosome 3), and miR-106b, miR-96, and miR-25 as an intronic cluster in the MCM7 gene on chromosome 7. MiR-155 in the provided example is located within the exon of the BIC gene on chromosome 21. MiR-483 provides an example of a miRNA located in the 3′UTR of the MeCP2 gene on chromosome 12 (**c**) Structural and sequence elements of pri-miRNAs and pre-miRNAs including a 5′ cap (m^7^G), a stem-loop configuration with localization of the Drosha-DGCR8 and Dicer-TRBP cleavage sites, and critical sequence features such as the seed region (nucleotides 2–8) that contains the critically important pivot at position 6, the 3′ supplementary region also known as delta seed. Enzymatic processing by Drosha and Dicer of the pre-miRNA leads to the release of a miRNA duplex containing the mature 5p strand and its complementary 3p strand, both of which have been found to potentially retain miRNA binding and regulating properties. (**d**) Hypothetical numerical representation of hsa-miR-375-5p isomiRs read alignments upon small RNA next-generation sequencing (NGS), which reveals heterogeneity in production of miRNA products for the hsa-miR-375-3p strand length, with base changes in both 5′ and/or 3′ regions due to nucleotide addition or removal during processing. Hypothetical distribution of the different isomiRs between normal and tumor tissue RNA. The length and sequence types of the different isomiRs highlight the biological complexity and regulation of miRNA processing, which can result in the shift of the different regions (i.e., seed, pivot, delta seed). (**e**) Mechanisms of miRNA-mediated gene silencing include perfect or imperfect seed matching with mRNA targets, leading to translational repression, mRNA deadenylation, decapping, degradation, or ribosome drop-off. (**f**) Intracellular miRNA biogenesis and trafficking of miRNAs, which are transcribed by RNA polymerase II as pri-miRNAs, cleaved by the Drosha-DGCR8 complex in the nucleus, exported via Exportin-5/RanGTP, and further processed in the cytoplasm by Dicer. Mature miRNAs are incorporated into the RNA-induced silencing complex (RISC) for target mRNA sequence recognition and repression or may be selectively loaded by RNA-binding proteins or other processes into multivesicular bodies (MVBs), for release into the microenvironment and/or circulation via EVs. (**g**) EV-mediated export of miRNAs and other RNA species. EVs such as exosomes and microvesicles carry diverse nucleic acids that include miRNAs, pre-miRNAs, tRNAs, circRNAs, mRNAs, other small non-coding RNAs, mtDNA, gDNA, etc. These vesicles facilitate intercellular communication, and the analysis of their content has the potential to reveal distinct biomarkers or biomarker signatures indicative of the biochemical state of specific cells. These vesicles can be found in diverse biofluids, transporting intact packaged RNA molecules and other important macromolecules.

MiRNAs utilize a standardized nomenclature system established by the miRBase database to ensure consistency across species and studies [24]. Mature miRNAs are named with the prefix “miR” followed by a unique identifying number (e.g., miR-1), while precursor miRNAs are denoted with the prefix “mir” in lowercase (e.g., mir-1). Letter suffixes (e.g., miR-1a and miR-1b) distinguish closely related family members with similar sequences, and a -5p or -3p suffix indicates the strand of origin from the 5′ (e.g., antisense) or 3′ arm (e.g., sense) of the precursor hairpin, respectively [25,26,27] (Figure 1a). Structurally, miRNAs are transcribed as long primary transcripts (pri-miRNAs) from a diversity of gene structures, including independently transcribed miRNA genes, miRNA clusters, and miRNAs intercalated within other genes (e.g., within exons and/or introns) or even within the 3′UTRs of other genes (Figure 1b). Once transcribed, miRNAs adopt a secondary single hairpin structure, prior to further processing (Figure 1c). Specific regions of the miRNA transcript enable their processing by protein complexes, ultimately leading to a single strand, which will be exposed for direct interaction with the 3′UTR of an mRNA transcript and regulation of gene expression.

Briefly, following transcription, the primary miRNA (pri-miRNA) located in the nucleus undergoes its initial cleavage, which is catalyzed by the microprocessor complex, composed of the RNase III enzyme Drosha and cofactor DGCR8, to generate a ~70-nucleotide precursor miRNA (pre-miRNA) (Figure 1d) [28]. This pre-miRNA forms an imperfect stem-loop structure, which is then exported to the cytoplasm by Exportin-5, a member of the importin-β family, in a RanGTP-dependent manner, where the RNase III enzyme Dicer processes it into a ~22-nucleotide miRNA duplex [29,30,31]. Following Dicer processing, the miRNA duplex is transferred to Argonaute (AGO) proteins, which form a core RNA-induced silencing complex (RISC) [32]. In humans, the AGO protein family comprises AGO1, 2, 3, and 4, with AGO2 being unique in its ability to mediate endonucleolytic cleavage of mRNA targets when near-perfect base pairing is present (e.g., near-perfect seed match). Most notably, this has been described for small interfering RNAs (siRNAs) [33,34,35]. Within RISC, one strand of the miRNA duplex, termed the guide strand, is selectively retained and anchored by AGO via the MID domain (i.e., binding the 5′ phosphate of the miRNA transcript) and the PAZ domain (binding the 3′ end of the miRNA transcript), while the complementary passenger strand is generally degraded. Once incorporated into RISC, where the miRNAs’ nucleic acids are unfolded and exposed, they are directed to their complementary target messenger RNAs (mRNAs) through sequence-specific base pairing, generally toward the 3′ untranslated region (3′UTR), to exert their inhibitory effect [36,37].

This miRNA/mRNA interaction is largely mediated by the degree of complementarity of the “seed” region located at the 5′ end of the miRNA (generally positions 2–8, with position 6, “the pivot”, being one of the most critical bases), which plays a critical role in target recognition (Figure 1c) [38]. It is important to consider that because the seed regions of miRNAs are vital for target recognition, even slight base variations can lead to differences in the recognition of their mRNA targets. This sequence variation can occur during Drosha or Dicer processing, which can result in the truncation or elongation of the 5′ or 3′ region, or both, of the sense and/or antisense miRNA transcripts, and leads to the formation of miRNA isoforms (i.e., isomiRs; Figure 1e) [39,40]. These sequence alterations can have profound effects on the miRNA function, as they shift the location of important binding regions (shifting positions 2–8 of the seed) involved in the recognition of binding proteins and physical exposure for access to their mRNA binding region. Due to these structural changes, isomiRs acquire the ability to regulate different mRNA targets compared to their canonical counterparts [41]. Consequently, studies have indicated that some isomiRs may have opposing effects within the same cellular context, wherein some result in the repression of a target gene’s expression, while others may fail to bind their target and/or may even promote gene expression indirectly by competing for binding sites or modulating the availability of the miRNA machinery [42,43]. This complexity adds an additional layer to the ability of miRNAs to regulate gene expression, which in turn influences diverse biological processes and disease states depending on the balance and abundance of specific isomiRs.

The binding of the miRNA-loaded RISC (miRISC) to the mRNA results in one of two possible outcomes, namely, translational repression or mRNA degradation (Figure 1f) [44,45,46]. In the case of partial complementarity (i.e., imperfect seed recognition), most often seen in animals, miRNAs will primarily repress translation by (i) inhibiting the initiation process, (ii) promoting deadenylation of the poly(A) tail, or (iii) blocking ribosomal progression [47,48,49]. This may ultimately lead to mRNA destabilization and degradation via decapping and exonucleolytic decay [50]. In contrast, when the miRNA-mRNA interaction displays near-perfect complementarity (i.e., perfect seed recognition), typically seen in plants, AGO-mediated endonucleolytic cleavage of the mRNA occurs, leading to its direct degradation [51,52]. While miRNAs are predominantly associated with gene silencing, under specific cellular contexts or in association with distinct RNA-binding proteins, some miRNAs have also been shown to enhance translation or stabilize target mRNAs, particularly during cell cycle arrest or quiescence, and some miRNAs have also been shown to interact with promoter regions of genes to enhance their expression [53]. These alternative, less common functions highlight the versatile and context-dependent nature of miRNA-mediated regulation within the cellular context.

## 3. MiRNA Deregulation and Potential Utility as Intracellular Biomarkers

The majority of miRNA genes are independently transcribed (Figure 1b), and as such, their expression is susceptible to a wide range of regulatory defects associated with disease. Most notably, this includes chromosomal translocations, mutations, copy number variations, methylation, and other transcriptional effects. Importantly, when the machinery of a cell is dysregulated, studies have confirmed that these alterations give rise to aberrant miRNA expression patterns that reflect not only the presence of a disease but also its subtype, grade, and stage [54,55,56]. For example, for breast cancer, distinct miRNA expression patterns have been reported that coincide with the different known molecular subtypes (i.e., triple negative, luminal A/B, HER2-positive) with high accuracy and reproducibility [57]. Subtype-specific miRNA patterns are often closely aligned with the hallmarks of cancer, including deregulation of proliferation, immune modulation, and epithelial–mesenchymal transition (EMT), further supporting the critical master regulatory role of miRNAs and their potential clinical relevance as biomarkers [58,59].

Importantly, miRNAs are highly stable in clinical specimens, including formalin-fixed paraffin-embedded (FFPE) tissues [60], needle biopsies, and liquid biopsies [61], due to their small size, GC-rich content, association with protein complexes like AGO, and transport by protective packaging within extracellular vesicles (EVs). This makes them ideal candidates for retrospective analyses and development of diagnostic assays using qRT-PCR, small RNA sequencing, NanoString assays, and/or locked nucleic acid (LNA)-based in situ hybridization technologies [62,63]. Tumor tissue studies have shown that stable miRNA expression patterns can be detected with high sensitivity and specificity to (i) identify tumor type, (ii) determine tissue of origin, (iii) assess metastatic potential, and (iv) predict treatment response [64]. Collectively, these features demonstrate the powerful capability of miRNAs as they regulate the genetic landscape of tumors.

Aside from their subtype-defining roles, specific miRNAs have been clearly shown to display oncogenic or tumor-suppressive characteristics in various cancer types. For example, in lymphoma, neuroblastoma, and non-small cell lung carcinoma, the miR-17–92 polycistronic cluster is frequently amplified, resulting in enhanced proliferation and inhibition of apoptosis through the targeting of PTEN and E2F1 tumor suppressors [65]. In contrast, in chronic lymphocytic leukemia, the deletion or epigenetic silencing of tumor-suppressive miRNAs such as miR-15a and miR-16-1 disrupts critical regulatory pathways, including those controlling apoptosis, cell cycle progression, and cellular senescence [66,67,68].

Therefore, reproducible alterations that result in disease-specific changes in the expression patterns of miRNAs have the potential to serve as robust biomarkers for screening assays. Reproducibly detectable miRNA expression changes are currently being evaluated for their potential to improve diagnosis, prognosis, and treatment response prediction across multiple malignancies. For example, miR-21, one of the most consistently upregulated miRNAs in cancer, is associated with poor prognosis and chemoresistance in breast, lung, and colorectal cancers [69], whereas high expression levels of miR-210, a hypoxia-responsive miRNA, correlate with tumor aggressiveness and radiotherapy resistance in breast and renal cancers [70]. Conversely, elevated expression levels of miR-34a and let-7 in lung, breast, and gastric cancers have been associated with improved relapse-free and overall survival [71,72]. Thus, given that deregulated miRNA expression, and particularly the expression of oncogenic miRNAs, can be correlated with specific malignancies and that some of these miRNAs can be encapsulated within extracellular vesicles (EVs) (Figure 1g) for intercellular communication, disease-specific miRNAs circulating in EVs are currently being investigated for their potential use in liquid biopsy (i.e., exhaled breath condensate (EBC), urine, saliva, sweat) analysis [73,74,75].

## 4. The Packaging of miRNAs for Extracellular Vesicle Transport

Although normal and diseased cell types will display different miRNA expression profiles/signatures that reflect functional changes, it has been shown that miRNA packaging into extracellular vesicles (EVs) does not fully reflect their cellular ratio [76]. Indeed, cellular miRNAs are selectively packaged into EVs via tightly regulated processes, which establish the type of control they can exert as intercellular communicators [77]. The selective loading of miRNAs into EVs is orchestrated by: (i) sequence motifs on the miRNAs, (ii) the subcellular localization of the miRNAs (i.e., cytoplasm, RNA granules, mitochondria, etc.), (iii) the post-transcriptional modifications of the miRNAs, (iv) the interactions of the miRNAs with RNA-binding proteins (RBPs), and (v) lipid-associated sorting machinery [78,79,80].

Increasingly, research has demonstrated the importance of “EXOmotifs”, which are short conserved sequences within miRNAs that facilitate recognition by RBPs. For example, miRNA sorting into intraluminal vesicles (ILVs), within multivesicular bodies (MVBs), is promoted through the binding of heterogeneous nuclear ribonucleoprotein A2/B1 (hnRNPA2B1) to GGAG motifs in a sumoylation-dependent manner [79]. Similarly, SYNCRIP (hnRNPQ) recognizes a conserved GGCU motif, aiding in the selective EV loading of miRNAs such as miR-3470a and miR-194-2-3p [80,81]. Y-box binding protein 1 (YBX1), a multifunctional RBP, plays a pivotal role in EV enrichment of oncogenic miRNAs such as miR-223 and miR-133 in breast cancer, lung cancer and glioblastoma, specifically under increased stress conditions [82,83].

Although Argonaute 2 (AGO2), the central component of RISC, was initially thought to chaperone miRNAs into EVs [84], refined isolation techniques have since shown that the majority of AGO2 proteins reside in non-vesicular ribonucleoprotein complexes [76]. These findings suggest that EVs carry a distinct pool of AGO2-free miRNAs, implying that active miRNA-sorting pathways can operate independently of canonical RISC activity [85]. Nevertheless, under oxidative stress conditions, AGO2 is phosphorylated and localizes to the MVBs, suggesting a context-dependent role in miRNA packaging [78,79]. Another example involves oncogenic KRAS signaling, which alters miRNA sorting by modifying AGO2 interactions and reprogramming RBP activity, ultimately leading to the preferential loading of specific miRNAs, such as miR-100, miR-10b, and let-7a, into EVs [86]. Importantly, recent studies have shown that AGO2 can facilitate N6-methyladenosine (m6A) modification of associated miRNAs, either by recruiting the methyltransferase complex (e.g., METTL3/METTL14) or by stabilizing miRNAs in a conformation accessible to methylation machinery. This AGO2-guided m6A labeling acts as a molecular signal for selective miRNA packaging into EVs, as m6A-marked miRNAs are recognized by specific m6A readers such as YTHDF proteins, which cooperate with RBPs to direct miRNAs into the EV pathway. For instance, the selective loading of miR-221/222 into exosomes, which promotes breast cancer metastasis, has been linked to AGO2-mediated recruitment of METTL3 and subsequent m6A modification [87]. In summary, while most miRNAs are incorporated into EVs through motif-specific sorting mediated by RBPs like hnRNPA2B1, SYNCRIP, and YBX1, AGO2 also indirectly contributes to EV-miRNA sorting by modulating cytoplasmic miRNA availability under altered cellular states.

Recent studies have identified vesicle-associated membrane protein-associated protein A (VAP-A), an endoplasmic reticulum (ER) protein, as a key regulator of miRNA loading into extracellular vesicles (EVs) [88,89]. VAP-A is localized at membrane contact sites between the ER and multivesicular bodies (MVBs), where it acts as a tether. At these sites, VAP-A coordinates the assembly of RNA–protein complexes and the miRNA-processing machinery, including Dicer and AGO2, thereby promoting the selective sorting of miRNAs into compartments involved in vesicle biogenesis [88]. Notably, Barman et al. demonstrated that VAP-A, in complex with its lipid transfer partner CERT, drives the biogenesis of RNA-containing EVs specifically at ER membrane contact sites [89]. This interaction promotes local lipid remodeling and compartmental recruitment of RNA-binding proteins, supporting a mechanistically distinct pathway of EV formation. Knockdown of VAP-A or CERT impairs the secretion of miRNAs such as miR-122 and miR-150 [90], whereas their overexpression enhances EV loading of pro-metastatic and immunomodulatory miRNAs, highlighting their functional importance in pathological states such as cancer.

Lipid metabolism critically influences EV biogenesis and miRNA cargo selection, particularly through the ceramide pathway [91]. Neutral sphingomyelinase 2 (nSMase2) facilitates ceramide production, and its inhibition reduces EV release, which alters their miRNA content and highlights the role of ceramide-rich microdomains for RNA cargo loading and vesicle budding [91]. This lipid-driven pathway functions independently of the endosomal sorting complex required for transport (ESCRT). In addition to VAP-A, components of the endosomal sorting complexes required for transport (ESCRT), such as Alix and TSG101, play critical roles in extracellular vesicle (EV) formation. They facilitate the budding of intraluminal vesicles (ILVs) within multivesicular bodies (MVBs) by interacting with membrane lipids and adaptor proteins, coordinating membrane deformation and scission events essential for EV biogenesis, and organizing the molecular machinery required for vesicle scission [92]. In contrast, the tetraspanin CD9 has recently been shown to couple ceramide-rich microdomains to YBX1-dependent miRNA sorting in a cholesterol-sensitive manner [93]. Several other tetraspanins, including CD63 and CD81, may also serve as scaffolding molecules that influence miRNA compartmentalization [94]. However, precise roles in facilitating miRNA sorting into EVs remain to be fully elucidated.

These diverse miRNA-sorting mechanisms are dynamically regulated by cellular stimuli, including hypoxia, oxidative stress, viral infection, and oncogenic transformation [95]. These stimuli alter both the expression and localization of RBPs and miRNA-modifying enzymes, leading to context-specific EV-miRNA signatures [96,97]. For example, García-Martín et al. demonstrated that distinct sequence codes guide small-RNAs into specific extracellular compartments, thus enabling selective communication across tissue barriers [98]. Altogether, a deeper understanding of these pathways is critical for elucidating how EVs orchestrate gene regulation in recipient cells, which holds great promise for advancing EV-based therapeutic strategies, especially for cancer, where EV miRNAs have been shown to serve as mediators of tumor progression (i.e., cellular education and establishment of the metastatic niche [99,100,101]).

## 5. Clinical Utility of miRNA Cargo Circulating in Cell-Specific EVs

Current research efforts are aimed at evaluating the diagnostic potential of miRNA profiles that circulate in cell-specific EVs released by diseased cells. Considering that diseased cells express different miRNAs than their normal counterparts and that their respective EVs also carry different miRNA cargos, there is a strong opportunity for developing EV isolation assays that enable selective enrichment of cell-specific EVs from diverse biofluids. Indeed, miRNA profile-based EV analyses have the potentially unique advantage of allowing detection and analysis of a tumor without having to directly access it. Indeed, as previously described, miRNA signatures may be useful for identifying the type, stage, and ability of tumors to respond to therapy. Therefore, major research and technological efforts have been invested in establishing state-of-the-art EV isolation assays before their analysis. Some of these efforts have been aimed at improving the specificity of EV isolation methods (i.e., size, density, and cell-specific marker selection) to enrich specific EV subpopulations most reflective of a particular disease state (e.g., cancer, inflammation, etc.) [102,103]. Although conventional bulk EV isolation methods, which include ultracentrifugation, size exclusion chromatography, density gradient selection, and precipitation, all offer opportunities to enrich and purify EVs, they cannot distinguish EVs based on their cellular origin [104,105]. Thus, several newer strategies that employ immunoaffinity-based capture assays and that utilize immobilized antibodies to recognize cell-specific surface proteins inherited by and exposed on EVs (e.g., EpCAM, PLAP, HER2) offer a more targeted approach for the selective enrichment of cell-derived EVs found in diverse biofluids [106,107,108,109]. An important consideration is that selecting the right biofluid is critical for yield, purity, and specificity; indeed, analyzing urine EVs may be more appropriate for detecting bladder cancer than EVs circulating in the bloodstream, for example.

Given the recognized limitations of conventional approaches, innovative platforms are being actively developed to improve specificity, reproducibility, and most importantly, scalability for the development of diagnostic assays. To this end, microfluidic and nanostructured platforms have been established to integrate size-based sorting with surface marker enrichment, thereby increasing precision whilst reducing the need for large/abundant sample inputs [110,111,112]. Among emerging translational approaches, the EvoLiver assay developed by Murla Bio represents a clinically relevant EV-miRNA profiling platform that integrates multi-marker EV capture with tissue-of-origin analysis to improve hepatocellular carcinoma detection [113]. Recent advances in extracellular vesicle (EV)-based diagnostics have highlighted the potential of tumor-derived EVs as highly informative liquid biopsy substrates for early cancer detection. Mercy BioAnalytics has developed a novel extracellular vesicle and particle (EVP)-based platform that detects the colocalization of tumor-associated biomarkers on individual EVs, rather than relying on single-analyte measurements, thereby improving both sensitivity and specificity for early-stage disease detection. Using combinations of ovarian cancer-associated markers such as BST2, FOLR1, and MUC1, this approach demonstrated improved discrimination between high-grade serous ovarian cancer (HGSC), benign ovarian masses, and healthy controls. More recently, application of this EVP-based blood test to preclinical UKCTOCS samples demonstrated 82.9% sensitivity for HGSC overall, 88.9% sensitivity for stage I/II disease, and 97.7% specificity, outperforming conventional CA125 testing in several metrics. These findings support the growing concept that multiplexed EV biomarker profiling may overcome some of the specificity and early-detection limitations associated with conventional circulating biomarkers in ovarian cancer screening [114,115]. Additionally, the drive towards the adoption of emerging techniques, such as tangential flow filtration, aptamer-based capture, fluorescence-activated EV sorting, acoustic or dielectrophoretic sorting, and other emerging technologies, is aimed at further enhancing throughput and selectivity [116,117,118,119].

Parallel to advances in isolation techniques for EVs, protein screening for the determination of their surfaceome offers a great avenue for elucidating how to identify unique surface biomarkers not only for diagnostic purposes but also for selective isolation of EVs (e.g., ExoPLA assay and surface-based proteomics assays) [120,121]. Importantly, specific miRNA profiling platforms and technologies such as next-generation sequencing (NGS), droplet digital PCR (ddPCR), and multiplexed qPCR panels have also been and continue to be optimized for their use in the analysis of low-abundance EV-derived miRNAs and other small non-coding RNAs [122,123,124,125]. These methods have already revealed that disease-specific EV-miRNA signatures can be remotely obtained from biofluids for a range of pathological conditions (e.g., COPD, Asthma, diabetes), but more particularly for cancer, where non-invasive screening and detection offer clinical advantages [126,127]. For instance, circulating EV miRNAs have shown utility in distinguishing malignant from benign lesions, monitoring treatment response, and predicting disease recurrence for breast, colorectal, lung and prostate cancer [128,129]. In several instances, the detection of EV-associated miRNAs outperforms the analysis of bulk free-circulating miRNAs in diagnostic performance, likely due to their protection from degradation, their selective intracellular packaging, and most importantly, the removal of non-specific sequences that create background noise, and in turn, due to their abundance, reduces sequencing depth and the formation of clusters for less abundant but more informative miRNAs and other small non-coding RNAs [130,131].

Building on these technological and clinical advances, a growing body of literature has identified specific EV-associated miRNAs with reproducible diagnostic, prognostic, and predictive value across multiple cancer types. While global miRNA profiling approaches provide broad signatures, several individual miRNAs have consistently emerged as robust biomarkers, particularly when enriched within EVs isolated from biofluids such as plasma and serum. Among the most extensively studied is miR-21, which is consistently upregulated in EVs derived from patients with breast, colorectal, and lung cancers and has been associated with poor prognosis and therapeutic resistance [132,133,134]. Similarly, miR-155 and miR-210, both linked to inflammatory and hypoxic tumor microenvironments, are frequently enriched in circulating EVs and correlate with aggressive disease phenotypes [135,136,137]. In pancreatic cancer, EV-associated miR-1246 and miR-25-3p have demonstrated particularly strong diagnostic performance, with reported area under the curve (AUC) values exceeding 0.90 in multiple cohorts, highlighting their potential for early detection [138,139,140].

In contrast, several tumor-suppressive miRNAs are consistently downregulated in cancer-associated EVs. For example, miR-34a, let-7 family members, and miR-486-5p are reduced in EVs from patients with lung and other epithelial cancers, reflecting loss of regulatory control over proliferation and differentiation pathways [71,141,142]. These opposing patterns of oncogenic and tumor-suppressive miRNAs further reinforce the functional relevance of EV cargo in shaping tumor progression and the tumor microenvironment. Notably, EV-miRNA biomarkers have also shown promise in more specialized contexts. In hepatocellular carcinoma (HCC), EV-associated miR-122 and miR-101 demonstrate high diagnostic accuracy, while decreased levels of miR-718 have been associated with recurrence risk [143,144]. In glioblastoma, microvesicle-associated miR-221 and miR-222 have been detected in circulation and linked to tumor burden, underscoring the importance of considering EV subtype when interpreting biomarker data [145].

Beyond differential expression of canonical miRNAs, emerging evidence indicates that miRNA isoforms (isomiRs) represent an additional layer of regulatory specificity with direct implications for EV biology and biomarker development [39,40,43]. IsomiRs arise from variations in Drosha/Dicer processing or post-transcriptional modifications, resulting in sequence heterogeneity at the 5′ and/or 3′ ends that can alter seed regions, secondary structure, and protein-binding affinity [39,40]. These subtle sequence differences have been shown to modulate interactions with RBPs such as hnRNPA2B1, YBX1, and AGO2, thereby influencing the efficiency and specificity of miRNA incorporation into EVs [79,80,81,82,84]. Notably, specific isomiR populations are often enriched in EVs relative to their canonical counterparts, suggesting active and selective sorting rather than passive release [39,40,43,98]. This selective enrichment has been linked to both 3′-end modifications (e.g., uridylation and adenylation) and 5′ seed shifts, which may alter motif recognition and downstream targeting capacity. As a result, isomiRs are increasingly recognized not only as functional regulators of gene expression but also as an additional layer of regulatory information that governs EV cargo selection [39,40,41,42,43,79,96,98]. Incorporating isomiR-level resolution into EV-miRNA analyses may therefore improve both the biological interpretation of intercellular signaling and the specificity of biomarker discovery.

Importantly, the majority of these studies rely on ultracentrifugation-based EV isolation, although alternative approaches such as polymer-based precipitation and immunoaffinity capture are also commonly employed. Variability in isolation methods, EV subtype classification (e.g., exosomes versus microvesicles), and cohort composition contributes to differences in reported biomarker performance. Additionally, the lack of standardized normalization strategies and limited incorporation of isomiR-level resolution may obscure biologically relevant signals and contribute to reproducibility challenges across studies. Nevertheless, many EV-associated miRNAs demonstrate AUC values ranging from ~0.75 to >0.90 across cohort sizes typically spanning between 50 and 200 patients, supporting their translational potential [138,139,140,143]. Collectively, these findings highlight that specific EV-associated miRNAs represent a functionally relevant and clinically actionable class of biomarkers. Their stability in circulation, combined with their disease-specific expression patterns and measurable diagnostic performance, positions them as strong candidates for integration into next-generation liquid biopsy platforms. A summary of representative EV-miRNA biomarkers, including their associated cancer types, directionality, clinical utility, and technical parameters, is provided in Table 1. For inclusion in Table 1, peer-reviewed articles were retrieved from PubMed, using combinations of keywords including “miRNA,” “extracellular vesicles,” “exosomes,” “biomarkers,” and “cancer.” Studies were prioritized based on (i) experimental validation of miRNA function or EV association, (ii) use of clinically relevant samples, and (iii) methodological rigor, including clearly defined EV isolation and RNA detection techniques. Only miRNAs with consistent directionality and demonstrated diagnostic, prognostic, or predictive value were selected.

## 6. The “Shadow” Side of miRNAs: Challenges in Therapeutic Translation

Despite the considerable promise of microRNAs (miRNAs) as both diagnostic biomarkers and therapeutic agents, multiple biological and technical challenges continue to hinder their successful clinical translation. Central to these limitations is the issue of efficient, stable, and targeted delivery. miRNAs are inherently susceptible to rapid degradation by circulating nucleases, and their small size facilitates renal clearance following systemic administration. As a result, achieving sufficient bioavailability at the intended target site remains a major obstacle. Furthermore, non-specific uptake by off-target tissues can dilute therapeutic efficacy while increasing the risk of unintended biological effects. To address these challenges, various delivery platforms have been explored, including lipid nanoparticles, viral vectors, and EVs [20,74,101]. Among these, EVs have generated particular interest due to their natural role in intercellular communication and their capacity to protect and transport RNA cargo in a biologically compatible manner [20,23,74,101]. However, the clinical application of EV-based delivery systems is still constrained by significant hurdles, including large-scale production, batch-to-batch variability, cargo loading efficiency, and incomplete understanding of biodistribution and targeting mechanisms [75,101,116]. Similarly, while synthetic nanoparticles offer advantages in terms of tunability and scalability, they may induce immune activation, exhibit toxicity, or lack precise tissue specificity [101,116]. Collectively, these limitations underscore the need for delivery systems that balance stability, targeting accuracy, and safety.

Beyond delivery, the intrinsic biological properties of miRNAs present additional complexities. A defining feature of miRNAs is their ability to regulate multiple mRNA targets simultaneously, often across diverse signaling pathways. While this pleiotropic nature can be advantageous in modulating complex disease networks, it also raises significant concerns regarding off-target effects and unintended pathway perturbations. Therapeutic modulation of a single miRNA may therefore lead to widespread transcriptomic changes, some of which may be detrimental or counterproductive. This lack of specificity complicates both the design and safety profiling of miRNA-based interventions.

Adding another layer of complexity is the context-dependent behavior of miRNAs. The functional role of a given miRNA can vary dramatically depending on cell type, tissue microenvironment, and disease state. As mentioned previously, in oncology, for example, certain miRNAs have been shown to function as oncogenes (oncomiRs) in one context while acting as tumor suppressors in another. This duality challenges the development of universal therapeutic strategies and necessitates a highly nuanced understanding of miRNA biology within specific disease contexts. It also highlights the importance of patient stratification and precision medicine approaches when considering miRNA-based therapies. Finally, standardization and reproducibility remain significant barriers across both diagnostic and therapeutic applications of miRNAs [75,124,150]. Variability in sample collection, RNA isolation methods, normalization strategies, and detection platforms can lead to inconsistent results across studies. This lack of harmonization complicates the validation of miRNA signatures and delays their integration into clinical workflows.

Taken together, these challenges emphasize that while miRNAs hold transformative potential in precision medicine, their clinical implementation requires continued advances in delivery technologies, improved targeting specificity, and deeper mechanistic insights into their context-dependent functions. Addressing these limitations will be critical for unlocking the full therapeutic and diagnostic value of miRNAs in human disease.

## 7. Bioinformatic Approaches to miRNA Analysis and Normalization Challenges

Advances in bioinformatics are essential for the analysis, interpretation, and functional annotation of miRNA sequencing data, particularly in the context of EV-associated miRNAs. The widespread adoption of high-throughput small RNA sequencing technologies has generated increasingly complex datasets, necessitating robust computational tools for accurate miRNA identification and quantification. Established platforms such as miRDeep, miRBase, and sRNAbench are widely used for miRNA discovery and profiling, enabling the detection of both known and novel miRNAs with high sensitivity [4,24,124]. These tools provide a critical foundation for downstream analyses, including differential expression and cross-sample comparisons.

Accurate prediction of miRNA function further relies on computational modeling of miRNA-mRNA interactions. Algorithms such as TargetScan, miRanda, and DIANA-microT leverage sequence complementarity, evolutionary conservation, and thermodynamic stability to identify putative targets and reconstruct regulatory networks [5,6,38]. While these approaches have significantly advanced the field, their predictive nature often results in high false-positive rates, underscoring the need for integrative frameworks that incorporate experimental validation and multi-omics data. Beyond miRNA identification and target prediction, recent efforts have increasingly focused on understanding the mechanisms governing selective miRNA loading into EVs. This process is now recognized as highly regulated rather than passive. Machine learning-based approaches have emerged as powerful tools for identifying sequence motifs, secondary structures, and molecular signatures associated with EV enrichment [79,80,81,96,98]. Features such as EXOmotifs and RNA-binding protein (RBP) interaction sites, including those recognized by hnRNPA2B1, YBX1, and AGO2, have been implicated in directing miRNA sorting into EVs [79,80,81,82,83,84,96,98]. As these models evolve, the integration of structural RNA features and contextual variables is improving the ability to predict EV-associated miRNA cargo with greater accuracy.

In parallel, integrative bioinformatic pipelines that combine small RNA sequencing with proteomic, transcriptomic, and EV characterization datasets are enhancing the resolution of EV-miRNA analyses. By linking miRNA profiles with EV surface markers, cellular origin, and protein cargo, these approaches enable more precise discrimination of biologically functional EV-associated miRNAs from background contaminants or passively released RNA fragments [74,75,102,106,120,121]. This is particularly important in biofluid-based studies, where technical noise and sample heterogeneity can obscure true biological signals. Such integrative strategies also contribute to improved reproducibility by promoting standardized analytical workflows and more rigorous data normalization. Despite these advances, a critical conceptual gap remains. While miRNA biogenesis, intracellular processing, and functional roles in disease have been extensively characterized, these processes are often studied independently of EV-mediated export mechanisms. There is currently no unified framework that links intracellular miRNA dynamics, including isoform diversity (isomiRs), RNA editing, and RBP-mediated regulation, with selective EV packaging and functional transfer. This disconnect limits the ability to interpret circulating miRNA profiles and to distinguish actively sorted, biologically relevant signals from passive RNA release, thereby constraining biomarker development.

A major barrier to progress is an emerging reproducibility crisis in EV-miRNA research, driven largely by the lack of standardized normalization strategies across studies [75,105,124,150]. Substantial variability arises from differences in pre-analytical and analytical workflows, including sample collection, biofluid handling, EV isolation methods, RNA extraction protocols, and sequencing platforms. In addition, contamination from non-EV sources, such as protein complexes and lipoproteins, further complicates data interpretation. Critically, there is no consensus on how to normalize EV-associated miRNA data. Current approaches, including normalization to input volume, EV particle number, total RNA content, or exogenous spike-in controls, each have inherent limitations and can yield markedly different results [62,75,120,122,150]. This lack of harmonization undermines cross-study comparability and hampers the validation of clinically relevant biomarkers. Collectively, these challenges highlight the need for a more integrated and standardized framework that bridges intracellular miRNA biology with EV-mediated transport mechanisms. Continued refinement of computational tools, combined with the establishment of robust experimental and analytical standards, will be essential for improving reproducibility and unlocking the full potential of EV-associated miRNAs as clinically actionable, non-invasive biomarkers.

## 8. Conclusions

Together, scientific discoveries and technological innovations are laying the foundation for EV-miRNA-based diagnostics. As such, continued optimization of isolation workflows, combined with advancements in ultrasensitive RNA detection and robust normalization strategies, is accelerating the clinical application of EV-miRNA profiling for minimally invasive detection of disease biomarkers. However, despite these advances, challenges remain in standardizing and precisely directing EV isolations to those precisely originating from diseased cells, miRNA normalization, and quantification for accurate measurements across laboratories. The field is moving toward harmonization efforts, including the use of spike-in controls, EV reference materials, and robust normalization strategies that have the potential to enable reproducible detection and clinical translation. As these technologies mature, the integration of machine learning algorithms for analyses of complex EV-miRNA profiles, possibly including isoforms, will further enhance the predictive power of liquid biopsy (i.e., exhaled breath condensates (EBCs), urine, saliva, tears, sweat) approaches for non-invasive EV-based disease diagnostics, particularly that of cancer. It is largely anticipated, however, that the integration of other known and unknown small RNA species has the potential to pave the way toward early and accurate disease detection, particularly at stages when treatments are most effective.

## Figures and Tables

**Table 1:** Validated EV-associated miRNA biomarkers in cancer.

miRNA	EV Source	Cancer Type	Regulation	Clinical Utility	EV Subtype	Isolation Method	Cohort Size	Reference
miR-486-5p	Plasma EVs	NSCLC	Down	Diagnostic	EVs	Ultracentrifugation	105	Cazzoli et al., 2013 [142]
miR-101	Plasma EVs	HCC	Down	Diagnostic	EVs	Ultracentrifugation	120	Sohn et al., 2015 [143]
miR-221	Glioblastoma microvesicles	Glioblastoma	Up	Diagnostic	Microvesicles	Differential centrifugation	~50	Skog et al., 2008 [145]
miR-222	Glioblastoma microvesicles	Glioma	Up	Diagnostic	Microvesicles	Differential centrifugation	~50–60	Skog et al., 2008 [145]
miR-1246	Serum EVs	Pancreatic	Up	Early detection	EVs	Ultracentrifugation	135	Matsumura et al., 2015 [146]
miR-196a	Plasma/Serum EVs	Pancreatic	Up	Diagnostic	EVs	Ultracentrifugation	120	Wang et al., 2009 [147]
miR-25-3p	Serum EVs	Pancreatic	Up	Early detection	EVs	Ultracentrifugation	150	Wu et al., 2025 [148]
miR-122	Serum EVs	HCC	Up	Diagnostic	EVs	Ultracentrifugation	200	Huang et al., 2010 [149]
miR-92a	Plasm EVs	Colorectal	Up	Diagnostic	Mixed EVs	Ultracentrifugation	90	Huang et al., 2010 [149]

## Data Availability

No new data were created or analyzed in this study. Data sharing is not applicable to this article.
